# Improving mammalian genome scaffolding using large insert mate-pair next-generation sequencing

**DOI:** 10.1186/1471-2164-14-257

**Published:** 2013-04-16

**Authors:** Sebastiaan van Heesch, Wigard P Kloosterman, Nico Lansu, Frans-Paul Ruzius, Elizabeth Levandowsky, Clarence C Lee, Shiguo Zhou, Steve Goldstein, David C Schwartz, Timothy T Harkins, Victor Guryev, Edwin Cuppen

**Affiliations:** 1 Hubrecht Institute/KNAW and University Medical Center Utrecht, Uppsalalaan 8, Utrecht 3584 CT, The Netherlands; 2 Department of Medical Genetics, UMC Utrecht, Universiteitsweg 100, Utrecht, 3584 GG, The Netherlands; 3 Life Technologies Inc., Advanced Applications Group, 500 Cummings Center, Beverly, MA, 01915, USA; 4 Laboratory for Molecular and Computational Genomics, Department of Chemistry, Laboratory of Genetics, UW-Biotechnology Center, University of Wisconsin-Madison, Madison, WI, 53706, USA; 5 Present address: Laboratory of Genome Structure and Ageing, European Research Institute for the Biology of Ageing; RuG and UMC Groningen, Antonius Deusinglaan 1, Groningen 9713 AV, The Netherlands

**Keywords:** Genome structure, Genome scaffolding, Mate-pair next-generation sequencing, Contig assembly, Rat genome

## Abstract

**Background:**

Paired-tag sequencing approaches are commonly used for the analysis of genome structure. However, mammalian genomes have a complex organization with a variety of repetitive elements that complicate comprehensive genome-wide analyses.

**Results:**

Here, we systematically assessed the utility of paired-end and mate-pair (MP) next-generation sequencing libraries with insert sizes ranging from 170 bp to 25 kb, for genome coverage and for improving scaffolding of a mammalian genome (*Rattus norvegicus*). Despite a lower library complexity, large insert MP libraries (20 or 25 kb) provided very high physical genome coverage and were found to efficiently span repeat elements in the genome. Medium-sized (5, 8 or 15 kb) MP libraries were much more efficient for genome structure analysis than the more commonly used shorter insert paired-end and 3 kb MP libraries. Furthermore, the combination of medium- and large insert libraries resulted in a 3-fold increase in N50 in scaffolding processes. Finally, we show that our data can be used to evaluate and improve contig order and orientation in the current rat reference genome assembly.

**Conclusions:**

We conclude that applying combinations of mate-pair libraries with insert sizes that match the distributions of repetitive elements improves contig scaffolding and can contribute to the finishing of draft genomes.

## Background

Genome assemblies consist of kilobase- to megabase-sized contiguous sequences of DNA (contigs) that need to be positioned in a correct order and orientation. This ordering of contigs (scaffolding) requires long-range structural information that reaches beyond the boundaries of contigs. Commonly used reference genome assemblies, like those of human [1,2], rat [3], and mouse [4], were all constructed using long-range structural information obtained by Sanger sequencing based applications. For example, mapped large insert clones (e.g., cosmid, fosmid and bacterial artificial chromosomes) and paired-end whole genome shotgun sequencing of plasmids with variable insert sizes contributed to elucidating the complexity of genomes at the structural level. Despite the high quality of these assemblies, tens to thousands of intercontig gaps still persist [3,5,6].

Currently, genomes are frequently sequenced by cost-effective next-generation sequencing (NGS) technologies. However, long-range structural information is often not available from such efforts and would require more costly and toilsome techniques than routine fragment or paired-end sequencing. The absence of long-range information poses significant challenges for dealing with repetitive sequences that often represent 50% of mammalian genomes [1,7]. Emerging technologies like long-read single-molecule sequencing [8] or single-molecule mapping systems like optical mapping [9-11], may eventually help to overcome many of the challenges put forward here. However, application of methods solely based on current NGS technology would be most optimal because such platforms are maturing fast and are very broadly available.Current NGS platforms are already capable of producing positional information using paired-end (PE) and mate-pair (MP) templates. PE sequencing involves the generation of pairs of sequencing reads derived from both ends of a contiguous DNA fragment. This sequencing modus is currently standard on most platforms but is limited by technology features (e.g. PCR constraints) that typically only allow for insert sizes of less than 500 bp [12]. MP sequencing, however, can provide much longer distance information [13], but requires several molecular sample processing steps to clone DNA fragment ends through a circularization step, making it a relatively laborious approach. Most commonly used MP approaches span 1 to 3 kilobase pairs (kb) and are therefore capable of spanning many repetitive or low complexity sequence elements. However, common repetitive elements [like LINE (L1) elements] in vertebrate genomes can span as much as 8 kb in size (Additional file 1) [7,14], illustrating the need for longer range information for comprehensive analysis of genome structures. To this end, various bioinformatic algorithms like CREST [15] and ALLPATHS-LG [16] have been developed to increase effective PE read span by systematically merging overlapping sequences. Experimentally, novel methods producing larger insert sizes have also been reported [17,18]. While these techniques clearly demonstrate the power of larger distance information, most do have limitations that could interfere with comprehensive analysis (e.g., maximum insert sizes of ~10 kb [17,19], potential biases introduced by enzymatic digestions [18], and relatively laborious or costly approaches that can only produce single fixed insert size libraries [20,21]). Furthermore, a systematic assessment of the utility and combination of different library insert sizes for resolving existing assembly difficulties in complex regions of genomes is currently lacking.

Here, we modified existing MP library construction protocols to allow for the generation of a wide range of small, medium and large insert size mate-pair libraries (3 kb up to 25 kb) and present a systematic comparison of their individual and combined utility for exploring mammalian genome structure. Our results show that two of the medium-sized MP libraries (8 kb and 15 kb) are most efficient for bridging repeats in the rat genome as well as for contig scaffolding. Furthermore, combining the medium-sized MPs with large insert (20-kb and 25-kb) libraries reduces the number of scaffolds by another 25% and results in a 3-fold increase in N50. Our results are useful to define the most optimal experimental paired-read approach to support the *de novo* assembly of mammalian genomes.

## Results

### Large insert mate-pair library generation

We constructed MP libraries through modification of the standard SOLiD protocol for mate-pair library construction (Additional file 2), to allow construction of MPs with insert sizes up to 25 kb. We used ~100 μg high-molecular-weight genomic DNA isolated from tissue of a single Brown Norway rat as starting material for all libraries. Sheared DNA was size-separated by pulsed-field gel electrophoresis [22,23] followed by excision of various fragment sizes from a single lane and conversion into mate-pair libraries. In total, we generated seven different library insert sizes, including six libraries produced with this adapted MP protocol and one PE fragment library that was prepared in a separate experiment (Table 1). Based on paired read mapping, the libraries showed median insert sizes of 170 bp (PE), 3 kb, 5 kb, 8 kb, 15 kb, 20 kb, and 25 kb.

**Table 1:** Sequencing and coverage statistics for all paired-read libraries

Library name*	**Sequenced pairs**	**Non-duplicate pairs (%)**	**Unique, consistently mapped pairs (%)**	**Median insert size (bp)**	**Physical coverage (x-fold)**	**5 M reads coverage (x-fold)**	PCR cycles **	Relative complexity ***	**inconsistent pairs forming clusters**	**inconsistent inter-chromosomal pairs forming clusters**
PE	160 M	151 M (95%)	131 M (87%)	166	8.5	0.34	5	>131 M	9.5%	2.0%
3 kb	17.7 M	16.3 M (92%)	15.2 M (93%)	3,208	50	6	14	4.6 M	24.0%	3.2%
5 kb_a	11.9 M	6.0 M (51%)	5.5 M (92%)	5,696	11	10.1	18	4.7 M	46.5%	3.7%
5 kb_b	16.7 M	14.7 M (88%)	14.0 M (95%)	5,811	28	10.1	13	>16.7 M	47.5%	4.6%
8 kb_a	20.8 M	8.6 M (41%)	7.8 M (91%)	8,293	25	16.2	14	5.7 M	58.4%	6.9%
8 kb_b	11.8 M	11.2 M (95%)	10.6 M (95%)	8,160	34	16.2	13	>11.8 M	50.6%	5.5%
15 kb_a	31.7 M	1.7 M (5%)	1.0 M (60%)	14,561	6	30.3	21	0.6 M	60.8%	7.6%
15 kb_b	11.6 M	1.6 M (14%)	1.2 M (73%)	13,556	7	30.3	21	0.7 M	23.2%	3.2%
20 kb	13.3 M	6.7 M (51%)	5.9 M (87%)	19,375	48	40.5	14	4.9 M	41.8%	4.7%
25 kb	56.9 M	2.3 M (4%)	1.1 M (49%)	25,871	11	50.6	17	0.7 M	51.9%	5.4%
TOTAL	352.4 M	220.1 M (62%)	193.3 M (88%)		228.5					

* The _b samples are retrieved from a replicate experiment using an independent DNA isolate from the same animal.

** Number of PCR cycles required to retrieve sufficient library molecules in the final adapter-mediated PCR.

*** Complexity is defined as minimal sequencing depth (in million clones) at which over half of the pairs are clonal.

**Figure 1:**
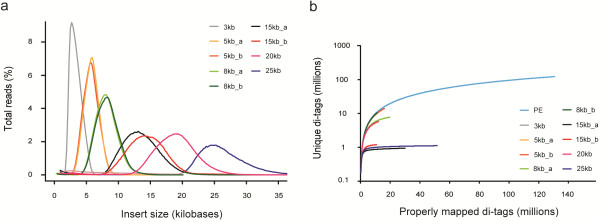
**MP insert size distribution and library complexity. **(**a**) Insert size distribution of all mate-paired libraries and biological duplicates. Data have been filtered for non-clonal pairs. (**b**) Complexity of each library is depicted by the number of unique read-pairs versus the number of properly mapped read-pairs. On the x-axis, increasing sequencing depth is represented based on actual sequencing data versus the amount of unique information obtained on the y-axis. A plateau indicates that a library has been sequenced to saturation.

To assess library complexity by determining the maximum number of unique reads obtainable from a MP library, two of the large-insert libraries (20-kb and 25-kb) were sequenced to a higher depth in an additional sequencing run. To assess reproducibility of the adapted MP protocol, three libraries (5-kb, 8-kb, and 15-kb) were generated in duplicate from independently isolated, sheared, and separated genomic DNA samples. Insert size distributions of the individually produced replicates were highly consistent (Figure 1a and Table 1). In total, 192.4 million pairs of MP reads and 160 million pairs of PE reads were generated. A total of 62.3 million non-duplicate MP read pairs and 131 million non-duplicate PE reads were consistently mapped against the rat reference genome, resulting in a genome-wide physical coverage of 228.5× (220× for MP libraries and 8.5× for 170-bp PE). Less than 1% of the paired reads were inverted (one of the reads in other orientation than expected) or everted (both reads in other orientation resulting in wrong order of tags) and approximately 10% were mapped remotely (i.e., to a distant genomic position, significantly deviating from what is expected based on the insert-size distribution). Remote, inverted, or everted events represent a mixture of 1) library construction artefacts due to chimeric molecules, 2) errors in the reference genome assembly (misassemblies) and 3) real structural differences between the reference strain and the substrain tested here. The first category typically involves stochastic events that are supported by a single read pair and that are filtered out by requiring multiple independent supporting read pairs for calling.

### Library sequencing and quality assessment

Sequencing libraries may suffer from low complexity due to library amplification steps in the protocol. When the proportion of unique library molecules is low due to inefficient molecular reactions or low amounts of input material, sequencing more reads of that same library would not yield any additional information, but only extra copies of previously sequenced molecules (duplicate reads). We assessed the complexity of each library by plotting the number of read-pairs with unique genome coordinates against the total number of all mapped pairs (Figure 1b). In general, the complexity of the small-insert libraries is higher than that of the large-insert libraries, which more quickly saturate to the level where deeper sequencing delivers predominantly non-informative duplicate reads. Duplicate reads do not necessarily affect the utility of the libraries, because these reads are filtered out as a first step in the analysis procedure; however, low complexity does decrease the capacity to obtain sufficient physical genome coverage. Three sample groups can be distinguished in Figure 1b: (1) high-complexity libraries that deliver approximately 100 million unique pairs (PE, 3kb, 5kb_b and 8kb_b), (2) medium-complexity libraries that result in about 10 million unique pairs (5kb_a, 8kb_a and 20kb), and (3) low-complexity libraries resulting in approximately 1 million unique pairs (15kb_a, 15kb_b, 25kb). Several of the low-complexity libraries show a plateau in the curve, indicating that these have been sequenced to saturation (25kb, 15kb_a). For others (5kb_b, 8kb_b), deeper sequencing would be informative.

Library complexity may be influenced by several experimental conditions. When starting with an equal quantity of genomic DNA, fragmentation for a standard PE library provides approximately 140-fold more unique molecules than for a 25-kb library. Furthermore, MP library preparation involves a circularization step (Additional file 2) that becomes less efficient as the size of the molecule increases. Quantification of DNA before and after circularization (and removal of non-circularized molecules) showed a circularization efficiency of up to 37% for libraries below 10 kb and 5–10% for libraries above 10 kb (Additional file 3). Each of these library generation steps has a negative impact on the recovery of material; for example, an input of 10 μg 25-kb size-selected DNA would result in approximately 6 ng (>4,000-fold reduction) of DNA for adapter ligation and subsequent adapter-mediated PCR. As a consequence, more PCR cycles are required for larger insert libraries to obtain sufficient amounts of library DNA for NGS (Table 1). Although the 3- and 5-kb insert libraries could routinely be generated at high complexity, we observed more technical variation for the large insert libraries. For example, the 20-kb library required only 14 PCR cycles during the library preparation procedure and performed well in the complexity analysis (comparable to 5- and 8-kb libraries). The 15- and 25-kb libraries required 21 and 17 cycles, respectively, and resulted in libraries of lower complexity (Figure 1b). These results indicate that the number of required PCR cycles is a very good predictive parameter for library complexity.

The 5-, 8-, and 15-kb libraries were generated in duplicate using DNA isolated from two different tissues of the same animal. The insert size distribution was found to be highly reproducible (Figure 1a), but the library complexity was much more variable between duplicates (Figure 1b). These differences might have been due to differences in DNA quality (e.g. amount of single strand breaks) or purity (e.g. associated protein or small molecule contaminants) of the DNA and subsequent differences in shearing efficiency. Indeed, DNA yields after size fragmentation were as much as 2.5-fold lower for the duplicate DNA sample (data not shown), which systematically resulted in less complex libraries. Most importantly, however, statistics for the amount of consistently mapped read pairs were comparable for all replicates (Table 1), indicating that the mapped unique read pairs were similar in quality (e.g., low chimaerism) and insert size. Low complexity in libraries could be circumvented by using larger amounts of input DNA and/or by optimization of shearing conditions to concentrate DNA in the desired size range. In our experiments we aimed for a broad size distribution to be able to simultaneously extract DNA for a range of different sizes.

Although the larger insert libraries come with more duplicate reads, far fewer sequencing pairs are required to physically cover the complete genome. It should be noted, that for all MP libraries in the experiments described here more than 10x physical coverage was obtained, including 48x coverage for 20 kb inserts.

To assess the value of the various insert size libraries for genome structure analysis, we determined the ability of each library to (1) physically cover the reference genome and overlap various repeat elements, (2) drive contig scaffolding, and (3) fix contig assembly issues in the current genome assembly (errors in contig order and orientation).

### Spanning repeats and physical genome coverage

**Figure 2:**
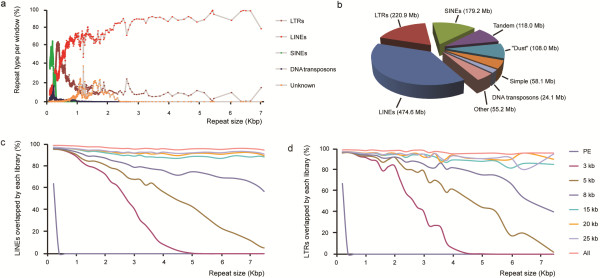
**Bridging of repeat elements by paired read libraries. **(**a**) The percentage of each repeat type per window of 1000 repeats (y-axis) is shown, relative to the size of each repeat on the x-axis. A higher density of dots indicates the presence of more repeats in the indicated size bin. (**b**) Pie chart of the largest classes of repetitive elements based on their total length (Mb) in the rat genome. Satellite repeats, RNA repeats, and low-complexity repeats are listed as “Other.” (c + d) Bridging by paired-tag libraries of all annotated LINEs (**c**) and LTRs (**d**) within contigs of RGSC 3.4. The size of LINE elements or LTRs (x-axis) is plotted against the percentage of elements of that specific size that were bridged by one or more read-pairs from each of the libraries. All single library datasets were normalized to 8.5× physical genome coverage.

The ability to physically cover a complete genome by sequencing is not only determined by the length of the read, the insert size of the library, and the number of paired reads, but also depends on genome-specific characteristics, like the composition and distribution of repetitive elements. The rat genome is representative for other mammalian genomes and contains 1.24 Gb of repetitive sequences, which is over 49% of the 2.51 Gb in the current reference genome assembly (RGSC 3.4, v.66 [24]). Retrotransposable LINE (L1) elements are the largest class of repeats with a total length of 474.6 Mb (18.9% of the genome), followed by retrotransposons that are flanked by long terminal repeats (LTRs; 220.9 Mb; Figure 2a and b). To evaluate the effect of library insert size on the degree of physical genome coverage, we merged data from duplicate libraries with the same insert size. Although the MP libraries had far more physical genome coverage, all datasets were normalized to an equal physical genome coverage based on properly mapped and oriented read pairs, which was limited to 8.5x by the available amount of data for the PE library. Next, we determined per library the fraction of bases per contig of the rat reference genome that is physically covered, specifically focusing on repetitive elements. Despite the same physical genome coverage and much higher base coverage, short-insert libraries (PE and 3 kb MP) were much less efficient in spanning long repetitive elements, such as LINEs or LTRs, than larger insert MP libraries (≥ 15 kb) (Figure 2c and d respectively). As expected, PE pairs overlapped hardly any of these elements but also the most widely used 3-kb MP libraries were found to only span approximately half of the 3-kb repeat elements, and only a few elements with sizes above 4 kb. Slightly improved results were observed for the 5-kb and 8-kb MP libraries, where approximately half of the repeats with a matched size could be spanned by at least one mate-pair. The 15-, 20-, and 25-kb libraries spanned over 90% of the repeat elements across the whole size spectrum and all displayed a very similar performance, indicating that there is limited added value for even larger insert sizes.

### Contig scaffolding

**Figure 3:**
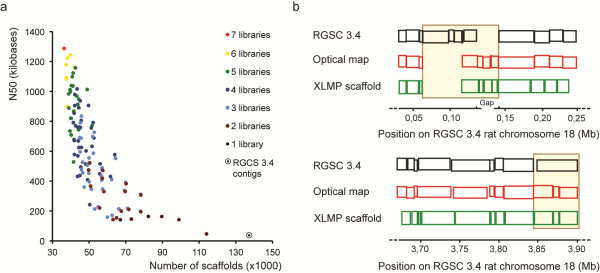
**Combinations of libraries with different insert sizes improve contig scaffolding. **(**a**) All library data sets were normalized to 8.5× non-clonal physical genome coverage resulting in the use of approximately 130 million pairs for the PE library to several million pairs for the MPs. The scaffold N50 (y-axis) as determined by SSPACE is plotted against the total number of scaffolds (x-axis) for each individual library and for all combinations of libraries. Scaffolding results for the current genome reference (RGSC 3.4) are displayed as well. (**b**) Representative examples of the genomic loci on rat chromosome 18 that show major discordance between optical map and the RGSC 3.4 reference genome. MP-assisted scaffolding restored concordance between sequence scaffolds and optical maps. The top panel (black) represents the reference genome assembly with the vertical lines indicating predicted SwaI sites; the middle panel (red) represents optical map data obtained using SwaI digests; the lower panel represents the rescaffolded genome using the MP data. The indicated positions on chromosome 18 are according to the current RGSC 3.4 assembly. A large region of approximately 75 kb (top panel) that shows low concordance with the predicted path of the optical map (0.065 Mb–0.14 Mb), increased significantly after MP-scaffolding. The bottom panel shows another example of increased resemblance to optical mapping data (3.85 Mb–3.90 Mb). Order and placement of contigs was shifted in the new scaffold resulting in SwaI sites identical to the optical map.

To evaluate the utility of the various libraries for guiding genome assembly, we simulated the scaffolding step of such process by using the 137,257 contigs from the current rat genome build and the different MP data sets as input for the SSPACE 2.0 [25] software. To allow for library insert-size comparison, we again used the normalized datasets at 8.5x physical coverage and determined the N50 (at least half of the genome bases are in scaffolds that are equal or exceeding the N50 value) and the number of scaffolds (segments of the genome reference consisting of contigs in known order, separated by gaps) for each individual library and combinations thereof, using the output of the SSPACE software (Figure 3a, Table 2 and Additional file 4). When we consider only the utility of single libraries, the N50 increases from ~38 kb for the PE data to 140–163 kb for the MP libraries of 5 kb and up. PE libraries are not effective in reducing the number of scaffolds as compared to the capillary sequencing-based contigs: a reduction of only 15 scaffolds is obtained (from 137,256 scaffolds in RGSC3.4 to 137,241 scaffolds using the PE data). In contrast, individual MP libraries decreased the number of scaffolds by up to more than 50% (~67,000 for the 5 kb library, which performs best of all individual MP libraries). When considering two insert size libraries, combination of 5 or 8 kb and 20 or 25 kb are most optimal with N50's of ~0.5 Mb. Intriguingly, 3 kb mate-pair libraries, which are most commonly used, showed the worst performance from all MP libraries, also when combined with other libraries. Including all libraries in the scaffolding process results in a further decrease of scaffolds (36,348) with an N50 increase up to 1.3 Mb. Increasing the physical coverage for a single insert library shows to be far less effective than combining libraries with different insert size (Additional file 5 and Additional file 6). For example, when we increase the coverage of the 5 kb insert library to 34x physical coverage, the N50 increases from 141 kb to 262 kb. However, combining 8.5x coverage data for the 5 kb insert library with similar coverage of any other MP library (up to 34x) results in much higher N50 values ranging from ~431 kb up to ~1 Mb.

**Table 2:** Scaffolding value of different paired-read library combinations

No. of libraries*	**Most efficient**	**Scaffold N50**	**Least efficient**	**Scaffold N50**
1	15 kb	163,475	PE	37,694
2	5 kb + 25 kb	522,027	PE + 3 kb	46,699
2	5 kb + 20 kb	474,308	PE + 5 kb	141,403
2	8 kb + 25 kb	470,890	PE + 25 kb	142,007
3	5 kb + 20 kb + 25 kb	834,964	PE + 3 kb + 5 kb	158,525
3	5 kb + 15 kb + 25 kb	789,954	PE + 3 kb + 8 kb	171,253
3	8 kb + 20 kb + 25 kb	726,289	PE + 3 kb + 25 kb	198,696
7	ALL	1,287,609	N/A	N/A

*All libraries were normalized to 8.5x physical genome coverage, limited by the amount of available data for the paired-end (PE) library.

### Reference genome improvement

Finally, we evaluated the value of various MP insert sizes for improving the existing rat genome assembly. To this end, we compared *de novo* scaffolds constructed using MP data with independently obtained genome-wide optical mapping data. Optical mapping is an integrated system that provides long-range genome structural information by the construction and analysis of genome-wide, ordered restriction maps [9,10,26,27]. We limited the analysis of concordance between sequence scaffolds and optical maps to one of the small rat chromosomes (RNO18), because the fine level optical structural alterations (OSAs) that were automatically called by the optical mapping pipeline [10] were manually curated between sequence scaffolds and optical maps, which required exploration on a case-by-case basis for mediation at the nucleotide-level. We divided the chromosome into 872 100-kb windows and found that 96 out of 872 of such bins harboured structural changes *within* scaffolds of RNO18 when comparing the MP-updated genome structure with the original genome. The 96 bins contained a total of 199 unique inconsistent connections between contigs within scaffolds. Next, we looked at structural differences *between* scaffolds of RNO18, based on the comparison of the MP-based scaffolding and the reference genome and observed many more bins to be affected (166/ 872 bins containing a total of 1374 inconsistent links between scaffolds). In total, 236 bins showed one or both types of inconsistent connections. Of these 236 bins, only 106 showed concordance with the reference genome. 130 bins were found to contain OSAs including absence or discordance of alignment between the optical maps and RGSC 3.4 genome assembly (detailed description in Additional file 7 and Additional file 8). Because the optical mapping system constructs ordered restriction maps and does not evaluate genome structure at the nucleotide level, not all discordances detected by the mate-pair analysis are revealed through optical mapping data. For example, small contigs or changes that do not overlap with a SwaI restriction site will not be identified.

We explored two of the largest segments with long-range disagreement between optical maps and RGSC3.4 assembly and conclude that MP-assisted re-scaffolding can recover concordance with the independently generated optical maps (Figure 3b). The complete MP data described here has therefore also been used for building the new genome reference of the rat (Rnor5.0, GenBank ID GCA_000001895.3, unpublished results).

## Discussion

Here, we show that large insert MP sequencing is a versatile tool for analysing genomes at the structural level and providing long-range information for genome scaffolding. Our results show that the addition of MP sequencing can dramatically increase contingency of mammalian genome references. In all analyses, insert sizes of >8 kb were shown to be essential because of their ability to bridge the longer and more abundant LINE and LTR elements. The analysis where the fraction of long repeats that is spanned by each MP library is determined shows that large insert MPs are capable of spanning ~90% of the annotated long repeats. The remaining approximately 10% of elements that could not be bridged by any of the MP reads can likely be explained by a highly repetitive nucleotide context around the repeat elements themselves. When a repeat element is surrounded by other repeats (mostly at centromeric or telomeric regions) one or both reads of the pair that would span such region can not be mapped uniquely to the genome and can thus not be included in the analysis. In agreement with this, our data show that even a combination of all libraries in this study fails to span 4–5% of repetitive elements larger than 3 kb in size (Figure 2c and d). Because rat and other vertebrate genomes contain tens of thousands of repeat elements that exceed the routinely used paired-end insert sizes (up to 500 bp), but include the very common LINE elements, we conclude that the inclusion of mate-pair libraries with insert sizes of 8 kb and above are instrumental for comprehensive reconstruction of genome structures.

The largest insert libraries (20–25 kb) were instrumental for increasing the N50 of scaffolds to megabase levels. Because the draft rat genome is already of relatively high quality, the improvements presented here have only mild effects. However, we anticipate that large insert MP sequencing will be very useful for finalizing low-pass capillary sequenced or NGS-based genomes like those of most primates as well as many of the vertebrate genomes. Genomes with large fractions or large segments of repeats, like that of the zebrafish or certain plants, might benefit even more from large insert mate-pair data as their genomes have a very high repeat content in combination with recently duplicated sequences. Furthermore, most ongoing genome sequencing projects employ next-generation sequencing techniques, and because *de novo* genome assembly based on short-reads is still in its infancy, contig sizes for vertebrate genomes are typically in the kilobase range [19,28,29]. Although paired-end data with insert sizes up to 500 bp are now commonly included in these processes, our results demonstrate that longer-range information as provided by the large insert MPs described here is essential for comprehensive genome assembly. It should be stressed that the structure of every genome of interest is unique and variable in complexity. Therefore, the optimal combination of MP insert sizes will vary as well. A quick examination of the repeat size and distribution could aid in determining which MP insert size combination is expected to be optimal, but experimental optimization or a broad range of libraries such as used here might be required.

In the analyses presented here, we focused on the application of large insert MPs for genome sequencing efforts, but the findings could be extrapolated to the detection of structural variation. Previous analyses of whole human genomes have shown that SVs affect more base pairs than single point mutations, yet the field has struggled to find a suitable approach for comprehensive detection of such events [30]. Hillmer et al. concluded that the most optimal insert size for SV detection is approximately 10 kb, although a thorough examination of the value of insert sizes above 10 kb was not described [17]. In unravelling the structure and organization of ultra-complex clustered mutation events, like the recently described chromothripsis, larger insert sizes (20–25 kb) may extend the detection limit and help to complete the overall picture [31-34]. It should be noted, however, that a “mate-pair only” approach also comes with disadvantages: small insertions, inversions, duplications, and deletions may be missed due to the broad size distribution and relatively low coverage at the base level.

Large insert MP sequencing represents a good alternative for the more traditional bacterial artificial chromosome-end sequencing because the sequencing libraries can be produced by relatively simple and scalable procedures without the need for laborious cloning and colony picking. Furthermore, the protocol can be fitted to all existing NGS platforms by changing the oligonucleotide adapters that are used. The mate-pair library construction protocol is relatively laborious compared to standard fragment library construction protocols, but with the latest improvements of the mate-pair protocol (SOLiD 5500 version), the procedure takes ~14 hours of hands-on work. More importantly, robustness of the protocol has been increased and the required input genomic DNA was reduced to only 1–5 μg for a standard ≤ 3-kb library, compared to 5–20 μg for the SOLiD V4 protocol (Additional file 9). The removal of column-based clean-up steps and the increased circularization efficiency (via the implementation of intra-molecular hybridization instead of circularization to an internal adaptor) are the main factors that allow for a reduced amount of input DNA. Nevertheless, our results show that limiting the amount of input DNA can strongly affect the complexity of the resulting library. For larger insert libraries it is therefore recommended to start with maximized amounts of DNA (>20 μg).

Although large insert MP libraries must be sufficiently complex, high physical genome-wide coverage is readily obtained at relatively low sequencing depth of tens of million read pairs. Alternative large insert approaches, like fosmid di-tag sequencing [20], have been documented to suffer from low library complexity, which may be overcome by using larger amounts of input material, but they have an additional disadvantage as they are restricted to a fixed insert size of approximately 40 kb [16,20,35,36]. Our data clearly demonstrate the added value of medium-sized insert libraries for genome structure analysis, a conclusion that was supported by Hampton et al. [20], who had to use supporting 4–6 kb mate-pair data to obtain essential long-range information that could not be obtained by fosmid di-tags alone. Using the MP protocol presented here, small, medium and large insert MP libraries can be generated in one go. Nevertheless, we did not generate libraries of equal size to 40-kb fosmid clones, so we could not determine if inserts of 25 kb are sufficient to fully replace 40 kb fosmid clones or if 40 kb pairs would span the last 4-5% of repeats that could not be covered by any of the MPs used here.

## Conclusions

We conclude that large insert MP sequencing provides a robust approach for comprehensive assessment of genome structure and for driving genome scaffolding processes. Applying combinations of mate-pair libraries with insert sizes that match the distributions of repetitive elements improves contig scaffolding and can contribute to the finishing of draft genomes. The sequencing platform flexibility, the scalable large insert size, the relatively limited amount of required sequencing tags, and the associated low sequencing costs make large insert MP sequencing an interesting and broadly applicable technique that is accessible for every routine sequencing lab.

## Methods

### Generation of MP libraries and mapping

To allow for the construction of large insert MP libraries, we modified the standard SOLiD 4 mate-pair library preparation protocol (Additional file 2). In short, genomic DNA was isolated from Brown Norway (BN/RijHsd) rat brain and testis tissue. DNA (100 μg) was sheared under mild conditions using HydroShear (JHSH204007, 20 cycles, SC15) and subsequently end-repaired (Epicentre End-It^TM^ DNA-end repair kit) in 1 mL End-It mix per 100 μg input DNA. CAP adapters were ligated in 500-μL reaction volumes of New England Biolabs Quick Ligase reaction mix. The amount of ligated adapter was determined based on the DNA content (100 pmol CAP adapter/pmol DNA). Following CAP-adapter ligation, DNA fragments were purified with phenol:chloroform:isoamylalcohol (PCI, pH7.9) by gentle mixing and centrifugation in MaXtract high-density tubes (QIAgen, 1.5mL, #129046). The fragmented DNA was separated via pulsed-field gel electrophoresis (PFGE; Bio-Rad CHEF Mapper XA system). PFGE conditions and settings were: 1% low melt agarose gel (Invitrogen, #16520100), 0.5× TBE, 14°C, 19 hours, forward current: 9.0 V/cm, switch time 0.08 s–0.46 s, reverse current 6.0 V/cm, switch time 0.08 s–0.46 s. Multiple size ranges (<7 kb, 7–10 kb, 10–14 kb, 14–18 kb, 18–24 kb, 24–33 kb, and >33 kb) were selected from the gel using a 1 kb extension ladder (Invitrogen, #10511-012). For unknown reasons, actual library insert sizes after library construction and mapping tend to be lower than their initial appearance on gel. A probable explanation for this is that most library construction steps may show a small bias towards smaller molecules (e.g. during circularization). Further on in the manuscript, each library will be referred to as the actual insert size as determined after data analysis. Following gel excision, DNA was carefully recovered using GELase^TM^ (Epicentre, #G09200). DNA fragments were circularized with a biotinylated internal adapter at a final DNA concentration of 1 nanogram per microliter (ng/μl). For every 40-μl reaction volume, 1 μl T4 DNA ligase was used. The reaction mixture was purified, and non-circularized fragments were removed by a plasmid-safe DNase treatment (Epicentre, #E3101K). Following linear DNA removal, DNA polymerase I-directed nick translation “pushed” the nick from the adapters into the circularized target DNA to generate sufficient tag length for sequencing (~100 bp for each tag, 13 minutes on ice water [0°C], inactivated with PCI). T7 exonuclease and S1 nuclease treatment were used to digest the circles at the position of the nick. The digested fragments of approximately 300 bp in size were end-repaired and bound to MyOne C1 streptavidin beads via the biotinylated internal adapter. Standard SOLiD P1 and P2 adapters were ligated to the blunt ends of the library molecules (a-tailing and alternative adapters should be used at this step to make the library compatible with the other sequencing platforms like Illumina, see Additional file 10), followed by another round of nick translation to remove the nick introduced by adapter ligation. Mate-pair libraries were amplified by PCR for 13–21 cycles, depending on the library. Amplification of the 14–18-kb size range samples (with an estimated final insert size of 10–12 kb) did not result in sufficient material (most likely because of unsuccessful adaptor ligation) and were not further included in the process. For all other insert size libraries we continued with templated bead preparation and libraries were successively sequenced on the SOLiD 4 system. For all libraries together, 192.4 million pairs of MP reads (AB/SOLiD V4, two slides) were sequenced. Paired reads were mapped against rat reference genome RGSC 3.4 using BWA v0.5.9 [37], and non-unique (based on identical read start sites for the forward and reverse read) and ambiguously mapped read pairs were removed from the data set.

### Generation of the paired-end library (170-bp) and mapping

For construction of the paired-end library (PE; 170-bp insert), the SOLiD 3 protocol for fragment library preparation was used (SOLiD™ 3 System Library Preparation Guide; Section 2.1). DNA (3 μg) derived from the Brown Norway rat was used for shearing using Covaris S2 (10 cycles of 60 s, intensity 5, 100 cycles/burst, 4°C). Sheared DNA was end-repaired using the Epicentre End-It^TM^ DNA-end repair kit. P1 and P2 adapters were ligated to the DNA fragments, and the library molecules were selected based on size (220–300 bp, including 90 bp for both adapters). PCR amplification was done for 5 cycles with primers specific to the adapters to obtain sufficient library molecules for ePCR and sequencing. Sequencing was done in paired-end mode (forward and reverse tag; 50 bp and 35 bp, respectively) on the SOLiD 3 system (1 slide AB/SOLiD V3). Approximately 1.6 × 10^8^ paired-end reads were sequenced (95% non-clonal) and mapped with BWA, resulting in a data set with a median insert size of 170 bp.

### Calculation of library insert size and contig scaffolding

Forward and reverse reads were mapped independently against contigs of rat RGSC3.4 genome assembly using BWA 0.5.9. Only read pairs with a single best hit for each tag (X0 flag equal to 1) were taken into consideration for estimate of insert size distribution. Analysis of library complexity was done by randomly sampling of reads from a library and determining the number of non-clonal pairs. Next, read pairs with exactly the same mapping coordinates of forward and reverse tags were marked as clonal and excluded from further analysis. Distribution of insert sizes was estimated from read pairs with proper orientation and distance between tags (below 100 kb).

To allow comparison of different library insert sizes for contig scaffolding, we created a subset of data for each library that corresponds to 8.5x non-clonal physical coverage of rat genome. We randomly sampled read pairs from each library, computing physical coverage represented by non-clonal pairs with expected orientation and distance between tags on chromosomal level (skipping pairs corresponding to first and last percentiles of insert size distribution). Read pairs from the normalized datasets with forward and reverse tags mapped to different contigs were selected for scaffolding analysis. Scaffolding was performed using SSPACE v2.0 software [25] with default parameters. The order in which the libraries were used by SSPACE was as recommended by the SSPACE manual - always from the smallest to largest insert size.

### Repeat analysis

Repeat annotation of the rat genome reference was obtained from Ensembl database [24] (v.66) and was used for calculation of size distribution and abundance of different repeat types. This annotation was used to determine the percentage of repeat elements spanned by mapped fragments from each mate-paired library. We used the normalized dataset where every library had 8.5x physical coverage (as described above). We considered only unambiguously mapped read pairs that had a proper orientation of tags and did not exceed 99^th^ percentile of fragment size distribution. Since individual copies of a mobile element or repeat class differ in size, we used 500 bp windows for calculation of percentage of mobile elements overlapped by fragments from each library.

### Comparison to optical maps

Original RGSC3.4 scaffolds and those obtained after NGS-assisted re-scaffolding were compared to each other by nucleotide BLAST search. Nearly identical (>99%) super-kb segments were plotted as Harr-plot visualization graphs. Most evident discordant regions were manually selected and the corresponding genomic segments were compared to optical maps [10] of Brown Norway rats. Optical maps are generated from large, randomly sheared high-molecular weight genomic DNA molecules that are stretched on a microscope slide. After stretching, the DNA molecule is digested with a SwaI restriction enzyme. While the DNA remains attached to the slide, the cuts become visible under the microscope as small gaps and the sizes of the stained DNA fragments can be measured. Multiple optical maps are then combined to form a comprehensive reference optical map for the Brown Norway rat genome. The optical maps used in this study are produced in the lab of David C. Schwartz and are available upon request. To compare our assembly with existing Brown Norway rat optical maps, nucleotide sequences of MP-enhanced scaffolds were digested *in silico* with a SwaI restriction enzyme. The *in silico* digested fragments were plotted next to optical maps, originally aligned to the RGSC 3.4 assembly and visually inspected for concordance.

### Availability of data

The data sets supporting the results of this article are available in the ArrayExpress repository under accession number E-MTAB-1082.

## Competing interests

EL, CL and TTH are employees of Life Technologies, manufacturer of SOLiD sequencing systems used in this study.

## Authors’ contributions

SvH, WK, NL, and EL, carried out the molecular experiments; FR, CL, SZ and VG performed bioinformatics analyses. SvH, WP SZ, SG, DS and VG performed and analyzed optical mapping experiments, SvH, WK, and EL generated large insert mate pair sequencing libraries, NL performed next-generation sequencing. SvH, WK, TH, VC and EC conceived of the study, and participated in its design and coordination and drafted the manuscript. All authors contributed to the final version of the manuscript and have read and approved it.

## Supplementary Material

Additional file 1 Size distribution of different types of LINE (L1) elements throughout the rat genome. Click here for file

Additional file 2 Schematic outline of the generation of large insert mate-paired libraries. Click here for file

Additional file 3 Table displaying the circularization efficiency of each individual library. Click here for file

Additional file 4 Scaffolding results of contigs from the current rat reference genome assembly using all possible combinations of paired-read libraries. Click here for file

Additional file 5 Combining insert sizes results in a more dramatic increase in N50 values than increasing only the physical coverage of one insert. Click here for file

Additional file 6 Combinations of insert sizes improve scaffolding N50 values more than increasing the coverage of a single insert library. Click here for file

Additional file 7 MP based scaffolding reveals inconsistencies with the current rat reference genome assembly. Click here for file

Additional file 8 Inconsistent links on rat chromosome 18 (RNO18) both within scaffolds and between scaffolds, based on a comparison between MP-based scaffolding and the current reference genome. Click here for file

Additional file 9 Comparison between the MP workflow of SOLiD 5500 and SOLiD V4. Click here for file

Additional file 10 Adapting the large insert MP protocol for Illumina sequencing. Click here for file
