# Supplementary material for: Improving mammalian genome scaffolding using large insert mate-pair next-generation sequencing
Source: BMC Genomics. 2013 Apr 16;14:257. doi: 10.1186/1471-2164-14-257 (PMC3648348; doi:10.1186/1471-2164-14-257)
Supplement: Additional file 1 — Size distribution of different types of LINE (L1) elements throughout the rat genome. [file 1471-2164-14-257-S1.pdf]

Additional Information to

Improving mammalian genome scaffolding using large insert mate-pair next-generation sequencing

Sebastiaan van Heesch[1], Wigard Kloosterman[2], Nico Lansu[1], Frans-Paul Ruzius[1], Elizabeth Levandowsky[3], Clarence Lee[3], Shiguo Zhou[4], Steve Goldstein[4], David C. Schwartz[4], Tim Harkins[3], Victor Guryev[1†]& Edwin Cuppen[1,2†*].

Additional Files 1-7

Additional File 1

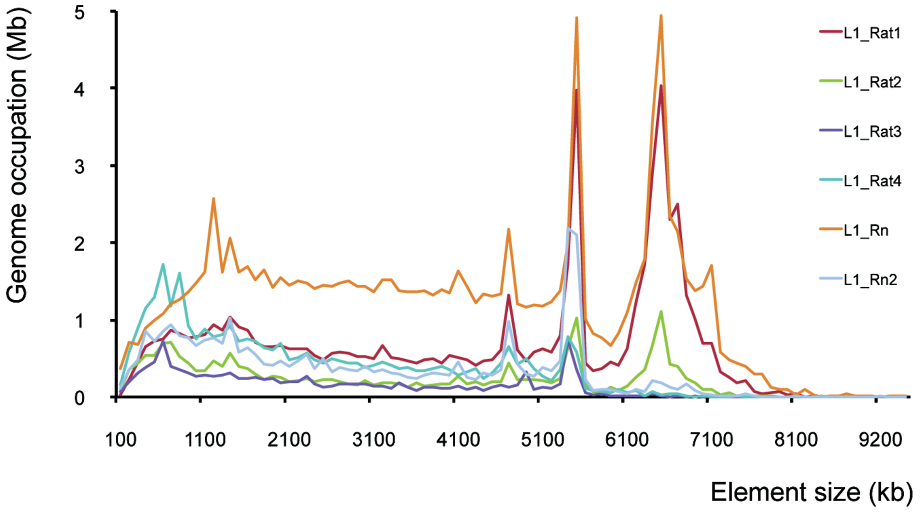

Additional file 1) Size distribution of different types of LINE (L1) elements throughout the rat genome. The length of six types of LINE retrotransposable elements present in the rat genome is plotted against the total size within the genome (Mb) that they occupy. While there are hundreds of thousands of small remains of incomplete LINE elements, the most frequent element sizes are 5.3 kb and 6.5 kb long. These are likely the youngest elements with largely intact LINE elements harboring a 5' UTR, one or two ORFs and a 3' UTR. All together, LINE elements encompass ~19% of the rat genome (475 Mb) making them the most abundant repeat element by total length (Fig. 2a), even though only few of them have retained their retrotransposable activity (Cordaux R, Batzer MA (2009) The impact of retrotransposons on human genome evolution. Nat Rev Genet 10: 691-703;Brouha B, Schustak J, Badge RM, Lutz-Prigge S, Farley AH, et al. (2003) Hot L1s account for the bulk of retrotransposition in the human population. Proc Natl Acad Sci U S A 100: 5280-5285).
