# Supplementary material for: Improving mammalian genome scaffolding using large insert mate-pair next-generation sequencing
Source: BMC Genomics. 2013 Apr 16;14:257. doi: 10.1186/1471-2164-14-257 (PMC3648348; doi:10.1186/1471-2164-14-257)
Supplement: Additional file 2 — Schematic outline of the generation of large insert mate-paired libraries. [file 1471-2164-14-257-S2.pdf]

Additional File 2

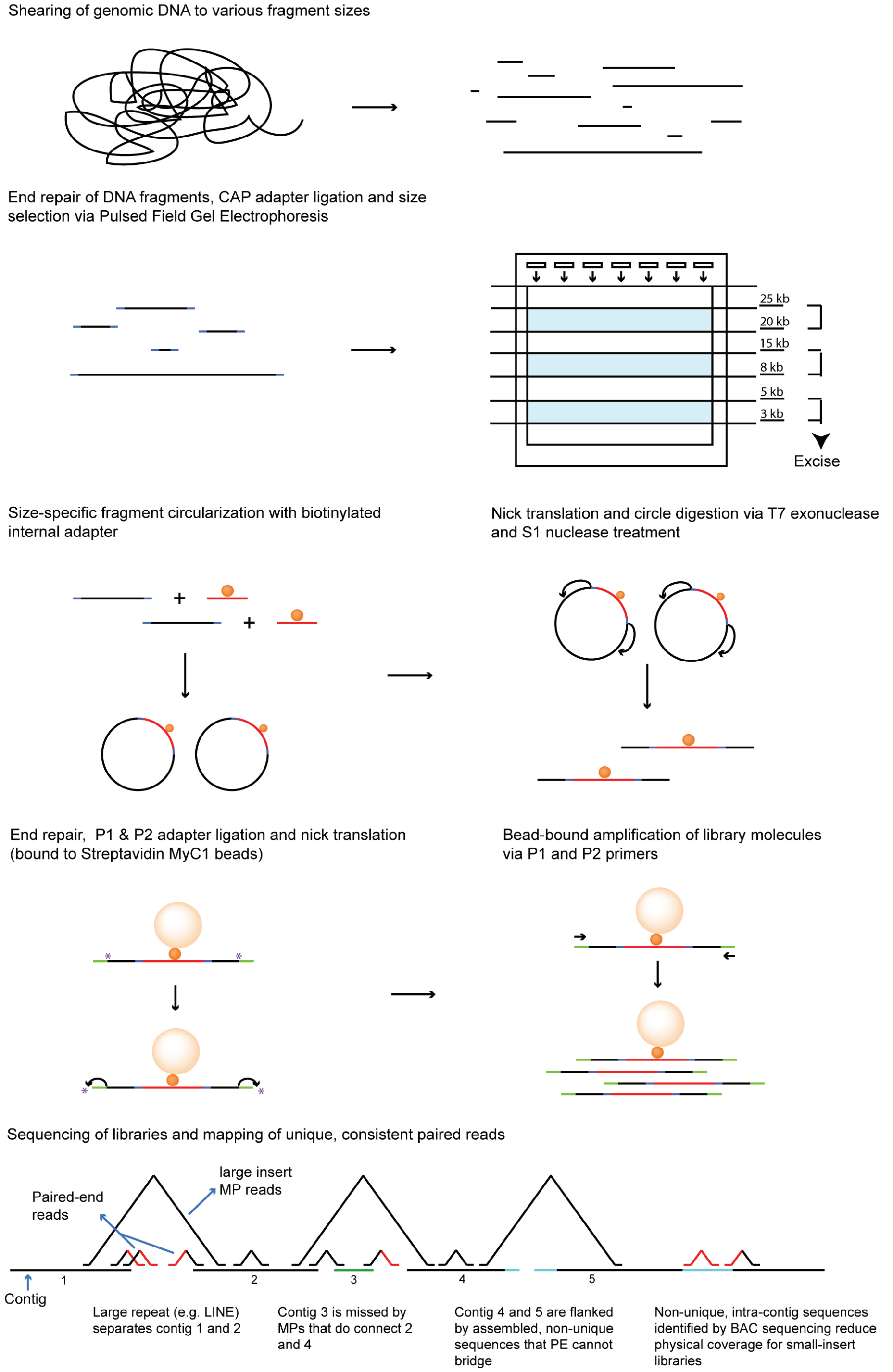

Additional File 2) Schematic outline of the generation of large insert mate-paired libraries. Step-by-step visualization of the large insert MP workflow. High molecular weight genomic DNA is sheared using Hydroshear. Sheared fragments are end-repaired followed by CAP adaptor ligation to the phosphorylated blunt ends. Next, all fragments are size separated

by pulsed-field gel electrophoresis. Depending on the shearing conditions, the desired size range(s) can be excised and recovered from the gel. In the experiments described here, 6 different size ranges were recovered. For each fraction, circularization was initiated at highly diluted concentrations to avoid the formation of chimeric molecules. The CAP adaptors of every molecule mediate circularization by binding a biotinylated internal adaptor with nucleotide overhangs. After ligation, which could only take place at one the two strands, the remaining single-stranded nick was translated into the DNA insert for approximately 100 bp. Next, T7 exonuclease and S1 nuclease were used to digest the circles at the nick-translated nicks. Finally, universal P1 and P2 adaptors were ligated to the remaining fragments and DNA fragments containing internal adapters were retrieved using Streptavidin beads and PCR amplified using P1 and P2 primers to retrieve full-length library molecules. These molecules were carefully quantified and analyzed by routine SOLiD sequencing. Bioinformatic tools are used to identify consistently mapped read pairs that bridging large gaps and known repetitive sequences.
