# Supplementary material for: Improving mammalian genome scaffolding using large insert mate-pair next-generation sequencing
Source: BMC Genomics. 2013 Apr 16;14:257. doi: 10.1186/1471-2164-14-257 (PMC3648348; doi:10.1186/1471-2164-14-257)
Supplement: Additional file 3 — Table displaying the circularization efficiency of each individual library. [file 1471-2164-14-257-S3.pdf]

Additional File 3

Library name	DNA quantity before circularization (µg)	DNA quantity after circularization (µg)	Circularization efficiency (%)
PE	3	no circularization	no circularization
3 kb	15.7	1.05	6.69
5 kb_a	1	0.37	37
5 kb_b	3	0.82	27.20
8 kb_a	2.05	0.56	27.07
8 kb_b	6	1.04	17.37
15 kb_a	10	0.5	5
15 kb_b	17	1	5.88
20 kb	17.5	1.64	9.35
25 kb	26.6	1.83	6.88

Additional File 3) Table displaying the circularization efficiency of each individual library. PE libraries have no circularization step. Smaller insert size libraries (e.g. 5 and 8 kb) have higher circularization efficiencies than the larger insert libraries (15, 20 and 25 kb).
