# Supplementary material for: Improving mammalian genome scaffolding using large insert mate-pair next-generation sequencing
Source: BMC Genomics. 2013 Apr 16;14:257. doi: 10.1186/1471-2164-14-257 (PMC3648348; doi:10.1186/1471-2164-14-257)
Supplement: Additional file 5 — Combining insert sizes results in a more dramatic increase in N50 values than increasing only the physical coverage of one insert. [file 1471-2164-14-257-S5.pdf]

Additional File 4

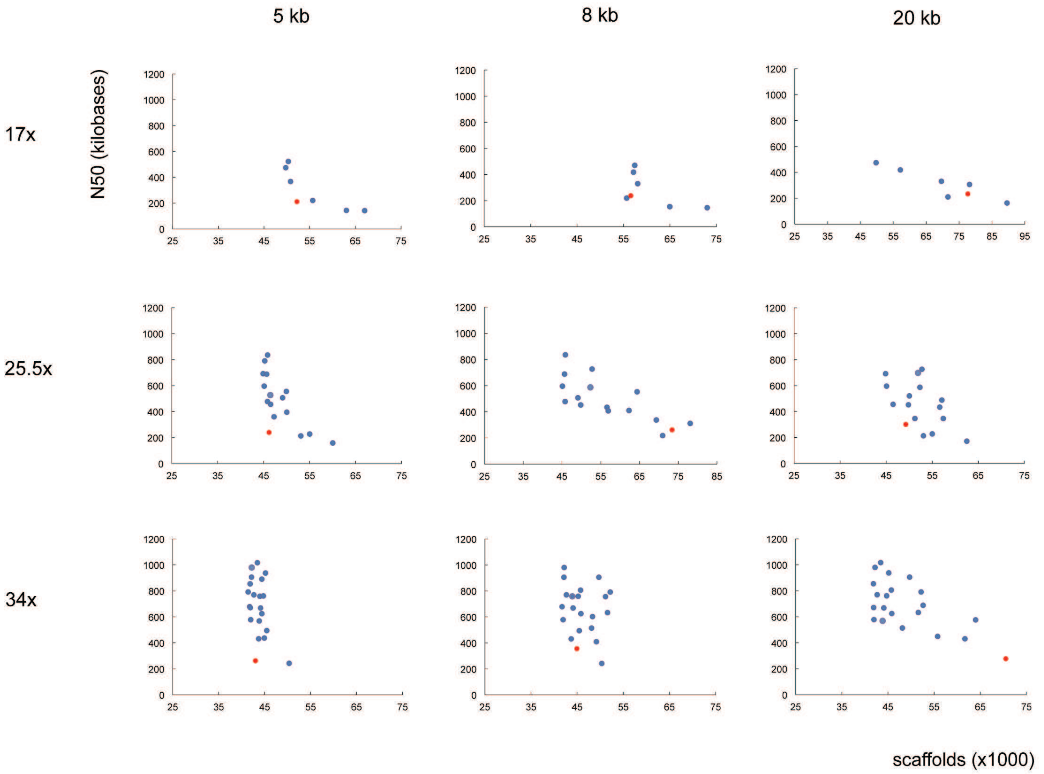

Additional file 4) Combining insert sizes results in a more dramatic increase in N50 values than increasing only the physical coverage of one insert. The effect of increased physical coverage (from 8.5x to 17x, 25.5x or 34x) during scaffolding using either a single insert (5 kb, 8 kb, 20 kb) or combinations of inserts is depicted (N50 on the y-axis, number of scaffolds on the x-axis). The results for single-insert libraries are depicted as red dots. The blue dots represent all other combinations of inserts that together make up 34x (8.5x per library; Additional File 9). For each insert, a clear increase in N50 is visible when combinations are used instead of solely increasing the coverage for that one insert. This supports a scaffolding approach that uses a range of insert sizes.
