# Supplementary material for: Improving mammalian genome scaffolding using large insert mate-pair next-generation sequencing
Source: BMC Genomics. 2013 Apr 16;14:257. doi: 10.1186/1471-2164-14-257 (PMC3648348; doi:10.1186/1471-2164-14-257)
Supplement: Additional file 7 — MP based scaffolding reveals inconsistencies with the current rat reference genome assembly. [file 1471-2164-14-257-S7.pdf]

Additional File 5

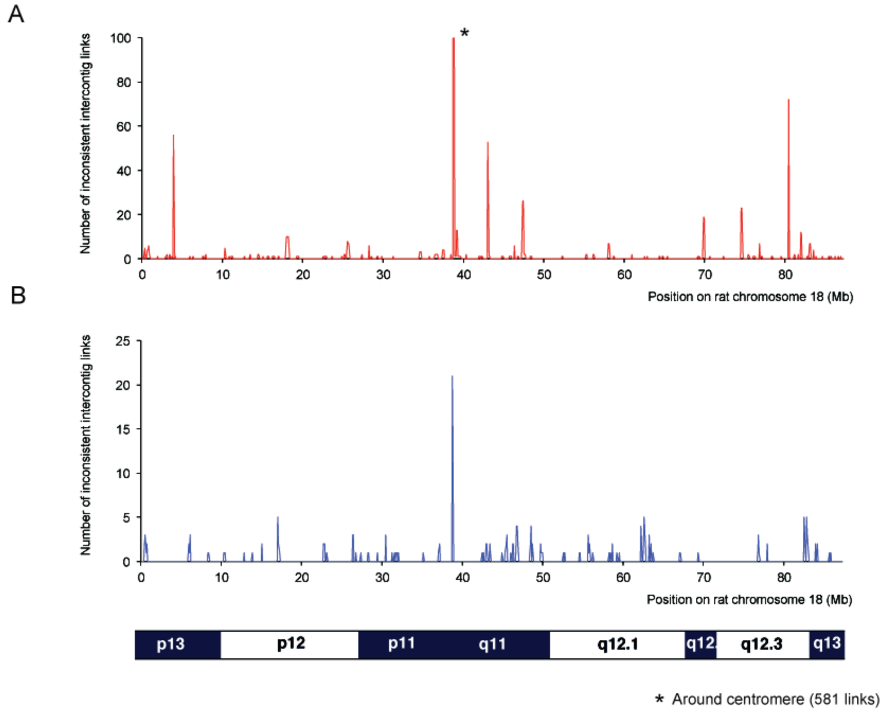

Additional file 5) MP based scaffolding reveals inconsistencies with the current rat reference genome assembly. Scaffolding of chromosome 18 (RNO18) was done based on the complete MP data set (3, 5, 8, 15, 20 and 25 kb libraries). (A) For each 100 kb bin, the number of inconsistent contig connections between scaffolds of chromosome 18 are displayed (with a minimum of 4 paired reads per link). Several regions with many discrepancies are identified, of which the largest is located around the centromere (marked by an asterisk at ~38 Mb). (B) Similar analysis as displayed in part A of the figure, but now only displaying the inconsistent contig links within a scaffold. The amount of inconsistent links for this latter category is much lower than between scaffolds, indicating that LMP data mainly improves longer distance connections. Please note that the Y-axes are differently scaled to improve the visibility of inconsistent links per category.
