# Supplementary material for: Improving mammalian genome scaffolding using large insert mate-pair next-generation sequencing
Source: BMC Genomics. 2013 Apr 16;14:257. doi: 10.1186/1471-2164-14-257 (PMC3648348; doi:10.1186/1471-2164-14-257)
Supplement: Additional file 9 — Comparison between the MP workflow of SOLiD 5500 and SOLiD V4. [file 1471-2164-14-257-S9.pdf]

Additional file 6

Workflow comparison SOLiD 5500 vs. SOLiD V4

SOLiD 5500	Shear and size-select DNA	End repair and MP adaptor ligation	Circularization[$], removal of linear DNA and nick-translation	Digestion of circularized DNA	dA-tailing, bead-binding and P1-T + P2-T adaptor ligation	Nick-translation and amplification (trial and final)
	~ 3 hr*	~ 2 hr	~ 2 hr	< 2 hr	~ 2 hr	< 3 hr
SOLiD V4	Shear and end repair	Ligate CAP adaptors and size-select	Circularization[$], removal of linear DNA and nick-translation	Digestion of circularized DNA	Bead-binding and P1 + P2 adaptor ligation	Shear and size-select DNA
	~ 2 hr	~ 3 hr*	~ 2 hr	< 2 hr	< 2 hr	< 3 hr

* 3 hr for 1-6 kb libraries. 30V O/N for 6-10kb libraries (0.5% agarose gel) or 19 hr for pulsed-field gel electrophoresis in case of >10kb libraries.

[$] For 5500, 4x more efficient intra-molecular hybridization is used compared to circularization to an internal adaptor in the V4 protocol.

Input comparison

SOLiD 5500	1-5 µg for 700bp to 3 kb insert sizes, 10-20 µg for 10 kb inserts.	Suggested input for 20-25 kb: 20-30 µg
SOLiD V4	5-20 µg for 600bp to 6 kb insert sizes	Suggested input for 20-25 kb: >30 µg

Additional file 6) Comparison between the MP workflow of SOLiD 5500 and SOLiD V4. The main differences are the changed circularization procedure and the A-tailing to allow P1-T and P2-T adaptor ligation. These two improvements result in more efficient reactions, thereby requiring lower amounts of input DNA, while the complete time of the protocol stays similar (~14 hours hands on).
