# Supplementary material for: Improving mammalian genome scaffolding using large insert mate-pair next-generation sequencing
Source: BMC Genomics. 2013 Apr 16;14:257. doi: 10.1186/1471-2164-14-257 (PMC3648348; doi:10.1186/1471-2164-14-257)
Supplement: Additional file 10 — Adapting the large insert MP protocol for Illumina sequencing. [file 1471-2164-14-257-S10.pdf]

Additional file 7) Adapting the large insert MP protocol for Illumina sequencing.

To apply this protocol for Illumina sequencing, an additional A-tailing step is required before adapter ligation and amplification. Where for SOLiD V4 sequencing P1 and P2 adapters are connected to the MP molecule using blunt-end ligation, Illumina (and SOLiD 5500) use A-tailing and adapters with a T-overhang. The standard A-tailing protocol for Illumina or SOLiD 5500 can be applied. The following are the adapters that need to be used for each platform (underlined = overhang).

SOLiD V4 adaptors:

P1

CCACTACGCCTCCGCTTTCCTCTCTATGGGCAGTCGGTGAT
TTGGTGATGCGGAGGCGAAAGGAGAGATACCCGTCAGCCACTA

P2

AGAGAATGAGGAACCCGGGGCAGTT
TCTCTTACTCCTTGGGCCCCGTC

CAP adaptor

CTGCTGTAC
GACGACA

Internal adaptor

CGTACATCCGCCTTGGCCGT
TGGCATGTAGGCGGAACCGG

SOLiD 5500 adaptors:

P1-T

CCACTACGCCTCCGCTTTCCTCTCTATGGGCAGTCGGTGAT
CCGGTGATGCGGAGGCGAAAGGAGAGATACCCGTCAGCCACT

P2-T

GAGAATGAGGAACCCGGGGCAGCC
TCTCTTACTCCTTGGGCCCCGTC

Illumina Paired-End Sequencing adaptors (# PE-102-1001)

(Oligonucleotide sequences © 2007-2012 Illumina, Inc. All rights reserved.):

PE1

ACACTCTTTCCCTACACGACGCTCTTCCGATCT
TGTGAGAAAGGGATGTGCTGCGAGAAGGCTAGp

PE2

pGATCGGAAGAGCTCGTATGCCGTCTTCTGCTTG
TCTAGCCTTCTCGAGCATACGGCAGAAGACGAAC

Roche GS-FLX Sequencing:

Use adapters from: GS-FLX Titanium General Library Preparation Kit (# 05233747001 - sequence unknown). As the molecule that results from the MP protocol in this paper is short enough for 454-shotgun sequencing, standard adapters can be used (paired-end is not necessary).
